# Regulatory Roles of the PI3K/Akt Signaling Pathway in Rats with Severe Acute Pancreatitis

**DOI:** 10.1371/journal.pone.0081767

**Published:** 2013-11-28

**Authors:** Ping Xu, Jing Wang, Zhi-wen Yang, Xiao-li Lou, Cheng Chen

**Affiliations:** 1 Department of Gastroenterology, Songjiang Hospital Affiliated the First People’s Hospital, Shanghai Jiaotong University, Shanghai, China; 2 Department of Pharmacy, Songjiang Hospital Affiliated the First People’s Hospital, Shanghai Jiaotong University, Shanghai, China; The Ohio State Unversity, United States of America

## Abstract

The phosphatidylinositol 3-kinase(PI3K)/protein kinase B (Akt) pathway plays a key role in inflammation. However, the regulatory roles of PI3K/Akt in severe acute pancreatitis (SAP) have not been elucidated. The aim of this study was to investigate the impact of wortmannin, a PI3K/Akt inhibitor, on SAP rats through exposure to sodium taurocholate (STC) after 3 h and 6 h. The SAP group was found to have a significant increase in pancreas Akt expression, along with the activation of serum amylase, TNF-α, IL-1β, and IL-6, and pancreas histological aggravation. The administration of wortmannin in SAP rats reduced Akt expression, attenuated the level of serum amylase and inflammation factor, and alleviated the damage of pancreatic tissue. Furthermore, the administration of wortmannin led to an obvious reduction in NF-κB and p38MAPK expression in SAP rats. These findings showed that the PI3K/Akt inhibitor wortmannin decreases inflammatory cytokines in SAP rats and suggests its regulatory mechanisms may occur through the suppression on NF-κB and p38MAPK activity.

## Introduction

Severe acute pancreatitis (SAP), an acute abdominal problem, is always accompanied by the impaired function or failure of multiple systems and multiple organs[1]. The onset and development of SAP are characterized by rapid changes, complicated illness states, and difﬁculty in treatment [2]. Approximately 25% of patients with acute pancreatitis will develop severe acute pancreatitis (SAP), with a mortality rate that reaches approximately 22.7% [3,4]. The pathogenesis of this disease is still poorly understood and has not been completely elucidated. Recently, experimental and clinical studies have demonstrated that acute pancreatitis (AP) is a common disease characterized by interstitial edema, digestive enzymes, acinar cell vacuolization, and the inﬁltration of neutrophils [5,6]. 

Our previous experiment [7] demonstrated that the prophylactic administration of pioglitazone attenuates the severity of SAP rats through inhibiting the p38MAPK and NF-κB signaling pathway. Recently, the phosphoinositide 3-kinase (PI3K)/protein kinase B (Akt) was known to be an endogenous feedback or compensatory mechanism involved in pro-inflammatory factor activation [8,9]. Inhibition of the PI3K/Akt pathway with a PI3K/Akt inhibitor decreased serum cytokine levels and increased the survival of mice subjected to sepsis [10]. However, it was still unclear whether PI3K/Akt could play an important role in SAP during the development of the disease. Thus, in the present study, we examined the *in vivo* regulatory roles of PI3K/Akt signaling pathway in SAP rats, and further explored its possible mechanism through evaluating the potential correlation between PI3K/Akt, p38MAPK, and NF-κB in inflammatory reactions associated with SAP.

## Materials and Methods

### Introduction of Severe Acute Pancreatitis

Seventy-two healthy male Sprague–Dawley rats, weighing 230-270 g, were purchased from the Animal Center of Shanghai Jiao Tong University of Chinese Medicine (Shanghai, China). Animals were housed in cages under a controlled temperature of 22±1°C and 12 h light–dark cycles, fed on standard laboratory chow with water *ad libitum*, and allowed to acclimatize for at least one week. The experiment was designed in accordance with the guidelines for the care and use of laboratory animals in research and was approved by the Ethical and Research Committee of Shanghai Jiao Tong University.

Sprague–Dawley rats were randomly divided into three groups: a sham operation group (SO), SAP model group (SAP), and wortmannin treatment group (W). Each group (n=24) was further divided into two subgroups based on the time points: a 3 h and 6 h subgroup (n=12 for each group). Surgical anesthesia was accomplished by intraperitoneal injection of 0.2% pentobarbital sodium. SAP was induced by retrograde infusion of 5.0% sodium taurocholate (1 ml/kg body weight) into the pancreatic and biliary duct. Half an hour before establishing the induced SAP model, rats were injected intraperitoneally with 1 mg/kg wortmannin. Rats in the sham group underwent a sham operation with nothing infused. Blood and pancreatic tissue samples were obtained at 3 h and 6 h after ductal infusion with sodium taurocholate. Blood samples were centrifuged at 3,000 g for 10 min. Serum was obtained and stored at -80°C until being assayed. Pancreatic samples were obtained, and a portion of the tissue was immediately frozen and maintained at -80°C for Western blot analysis and real-time PCR. The others were ﬁxed in 10% formaldehyde for histopathological examination. 

### Serum amylase activity and inﬂammatory cytokine assay

The amylase activity of each sample was measured with an automated biochemistry analyzer. TNF-α, IL-1β, and IL-6 levels in sera were assayed with ELISA kits (Neobioscience, Beijing, China).

### Histopathological examination

The pancreatic tissue from each rat was incised and fixed in 10% neutral-buffered formaldehyde, embedded in paraffin, and stained with hematoxylin and eosin. Edema, inflammation, as well as hemorrhage and necrosis of the pancreas were each graded from 0 to 4 as described below: edema (0 = no edema, 1 = focally increased between lobules, 2 = diffusely increased between lobules, 3 = tense acini, widely separated lobules, and 4 = gross lobular separation); inflammation (0 = no leukocyte, 1 = leukocyte 2-10/HP, 2 = leukocyte 11-20/HP, 3 = leukocyte 21-30/ HP, and 4 = leukocyte >30/ HP); hemorrhage (0 = no hemorrhage, 1 = blood in 25% parenchyma, 2 = blood in 25-50% parenchyma, 3 = blood in 50-75% parenchyma, and 4 = blood in 100% lobules); necrosis (0 = no necrosis, 1 = periductal parenchymal destruction, 2 = focal parenchymal necrosis, 3 = diffuse loss of lobules, and 4 = severe loss of lobules). The pathological scores presented in the study were obtained through the addition of these different scores. 

### Western blot analyses

Western analysis was used to investigate Akt, p38MAPK, and NF-κB p65 activation in pancreatic tissue. The pancreatic tissue from each rat was homogenized in ice-cold lysis buffer containing protease inhibitors. After removing cell debris by centrifugation, proteins were separated by 10% sodium dodecyl sulfate-polyacrylamide gel electrophoresis and electrophoretically transferred to nitrocellulose membranes. Nonspecific binding was blocked overnight at 4°C by 3% bovine serum albumin. After blocking, membranes were incubated with anti-p38/p-p38, anti-Akt/p-Akt and anti-NF-κB p65/p-NF-κB p65 antibodies for 2 h at 25°C. Subsequently, membranes were washed twice and incubated with anti-rabbit IgG Fc (HRP) secondary antibodies (Biotech, Taiwan, China) for 1 h at 25°C. 

### Real-time PCR

Real-time PCR was used to investigate the expression of Akt, p38MAPK, and NF-κB p65 mRNA in pancreatic tissue. Total RNA in pancreatic tissue was isolated with TriReagent (Invitrogen, California, American) according to the manufacturer’s instructions. The reaction mixture was amplified in a DNA thermal cycler (Bio-Rad, American) and the incubation and thermal cycling conditions were as follows: denaturation at 95°C for 30 s, annealing and extension at 60°C for 34 s. The number of cycles was 40. The primer sequence for β-Actin was: sense 5’-CCTGGCACCCAGCACAAT-3’, antisense 5’-GGGCCGGACTCGTCATAC-3’, and the expected length 105 bp. The primer sequence for Akt was: sense 5’-TCACCTCTGAGACCGACACC-3’, antisense 5-ACTGGCTGAGTAGGAGAACTGG-3, and the expected length 121 bp. The primer sequence for p38MAPK was: sense 5’-CCTTGCCACTTTGGCTTCTC-3’, antisense 5’-AGCAGCCTCTCTGTCACTGA-3, and the expected length 202 bp. The primer sequence for NF-κB was: sense 5’-AATGGCGATCTGGGTGTCC-3’, antisense 5’-CCTTGCGGGTCAACTTGTAGA-3’, and the expected length 182 bp (Sangon Biotech, Shanghai, China ). 

### Statistical analysis

Results were expressed as mean ± standard deviation (SD). All data were analyzed by a factorial design ANOVA test using SPSS 13.0 with statistical significance set at P < 0.05.

## Results

### Histopathological examination and serum amylase activity

**Figure 1:**
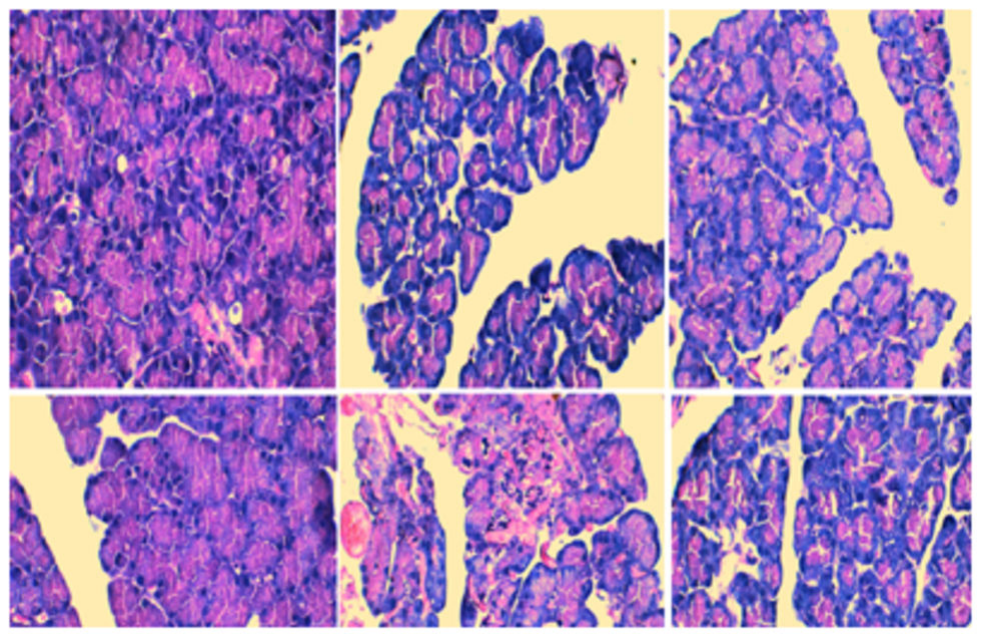
Histological examination of pancreas in each group at the 3 h and 6 h time point (original magnification, 400x). A: SO group, observed at 3 h time point; B: SAP group, observed at 3 h time point; C: W group, observed at 3 h time point; D: SO group, observed at 6 h time point; E: SAP group, observed at 6 h time point; F: W group, observed at 6 h time point.

**Figure 2:**
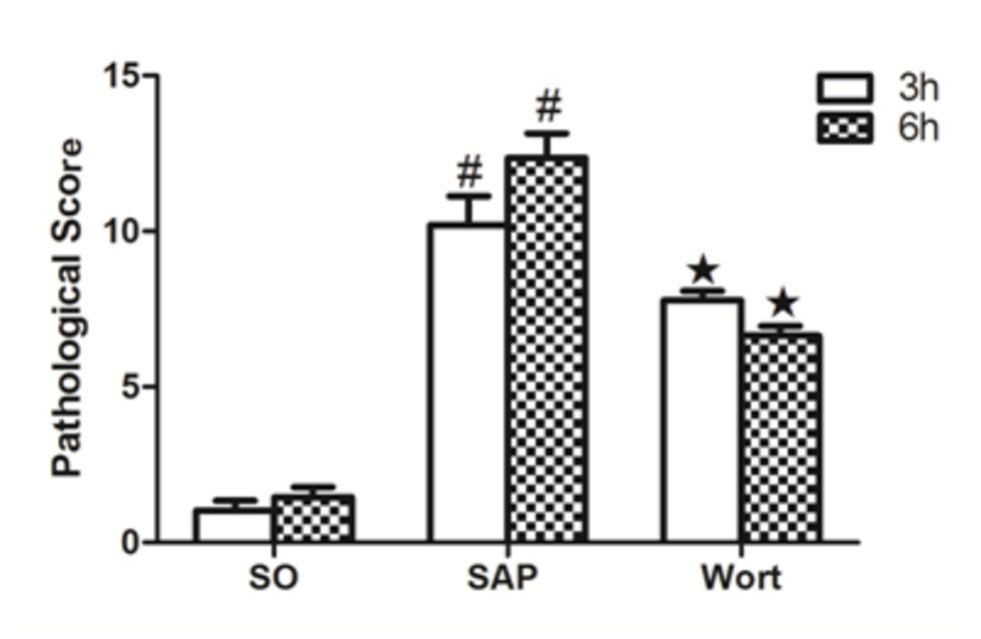
Pathological scores for the pancreas in each group at the 3 h and 6 h time point. ^*#*^ *P*<0.05 vs. SO group; ^★^ *P*<0.05 vs. SAP group.

 As shown in Figure 1 and Figure 2, histological changes and pathological scores for pancreatic tissues markedly changed from the control rats. Compared with the SO group, pancreatic injury in SAP rats was characterized by tissue edema, leukocyte infiltration, acinar cell necrosis, and hemorrhage at the 3 h point. As the duration increased, the pancreatic injury was aggravated and the pathological scores were significantly elevated (Figure 1D, E, P < 0.01). Treatment with wortmannin obviously ameliorated the severity of inflammatory response in SAP animals, decreasing pancreatic injury at 3 h and 6 h, respectively (Figure 1C, F , P < 0.05 at 3 h points, P < 0.05 at 6 h points).

**Figure 3:**
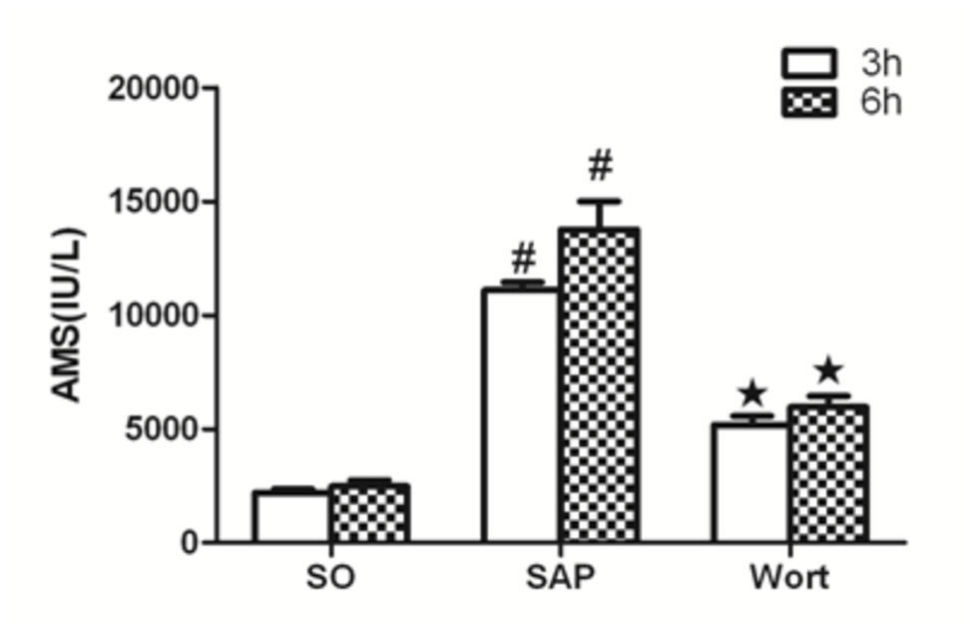
Serum amylase in each group at the 3 h and 6 h time point. ^*#*^ *P*<0.05 vs. SO group; ^★^ *P*<0.05 vs. SAP group.

Serum amylase activity from the control rats is shown in Figure 3. In accordance with the histopathological examination, serum amylase activity in SAP rats significantly increased compared to the SO group. The wortmannin treatment group had markedly decreased levels of serum amylase compared to the SAP rats. 

### Inflammatory cytokine levels in sera

**Figure 4:**
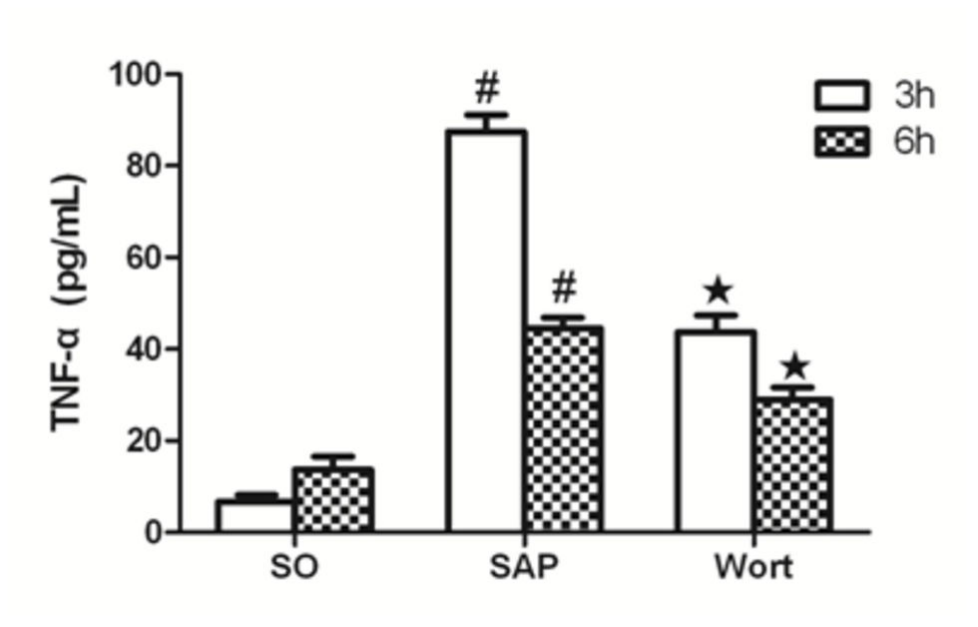
TNF-α level in each group at the 3 h and 6 h time point. ^*#*^ *P*<0.05 vs. SO group; ^★^ *P*<0.05 vs. SAP group.

**Figure 5:**
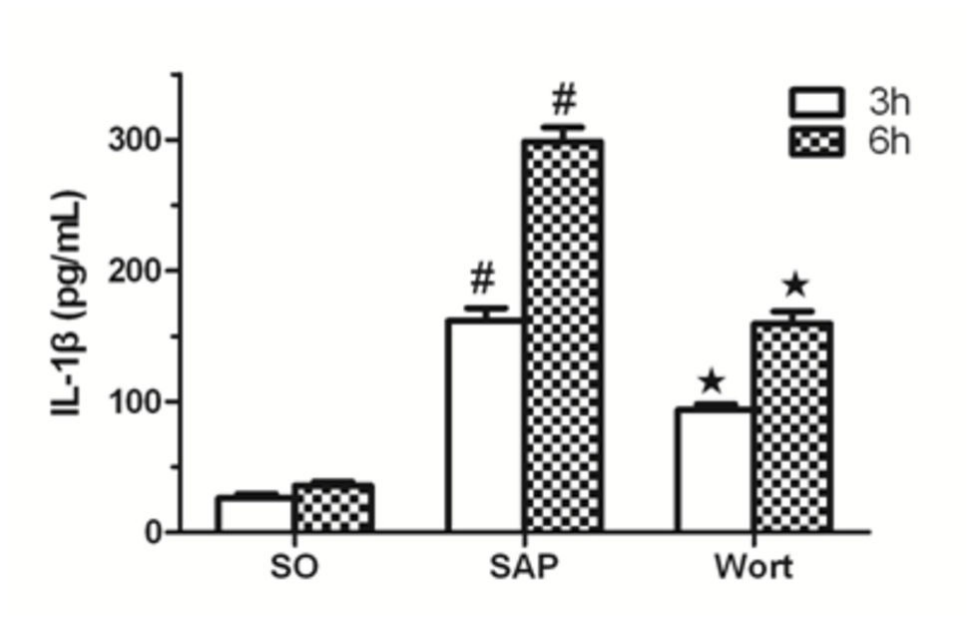
IL-1β level in each group at the 3 h and 6 h time point. ^*#*^ *P*<0.05 vs. SO group; ^★^ *P*<0.05 vs. SAP group.

**Figure 6:**
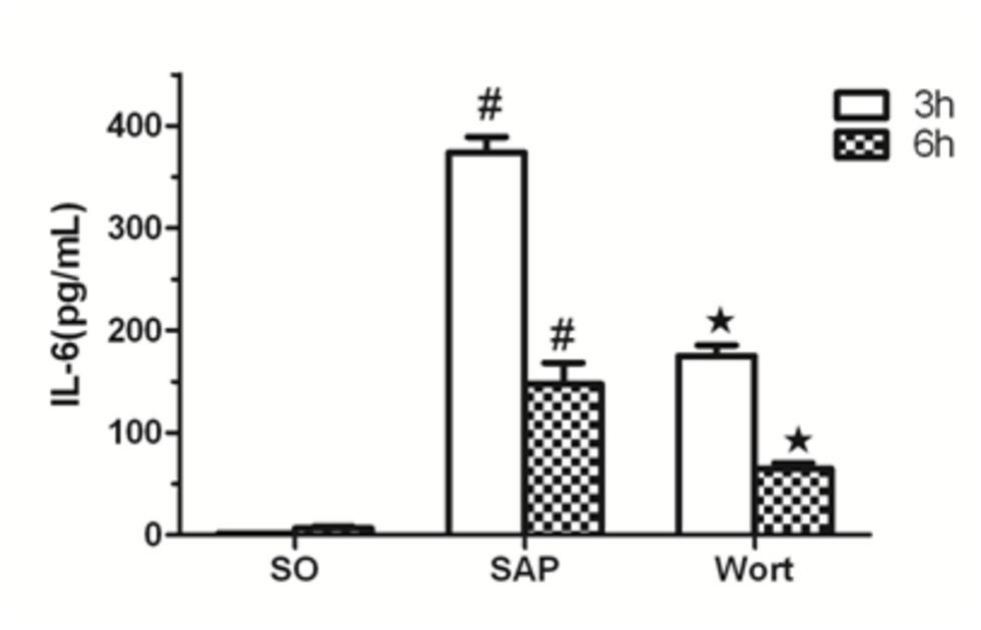
IL-6 level in each group at the 3 h and 6 h time point. ^*#*^ *P*<0.05 vs. SO group; ^★^ *P*<0.05 vs. SAP group.

The measurement of serum inflammatory cytokine levels showed that SAP rats had an increased expression of the pro-inflammatory cytokines TNF-α, IL-1β, and IL-6 (Figure 4, Figure 5 and Figure 6). In addition, the level of TNF-a and IL-6 in SAP rats increased and reached a peak at 3 h after pancreatitis was induced, then gradually decreased. In contrast, these levels were reduced by treatment with wortmannin. These resultes indicated that wortmannin, as a PI3K/Akt inhibitor, could effectively attenuate pancreatic injury 

### Akt, p38MAPK, NF-κBp65 mRNA expression

**Figure 7:**
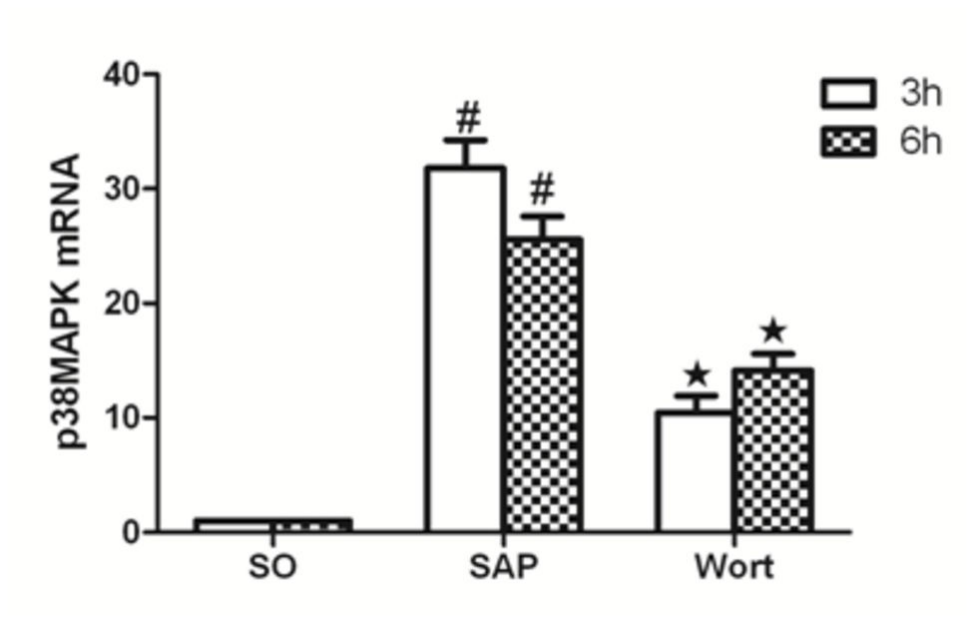
p38MAPK mRNA expression in each group at the 3 h and 6 h time point. ^*#*^ *P*<0.05 vs. SO group; ^★^ *P*<0.05 vs. SAP group.

**Figure 8:**
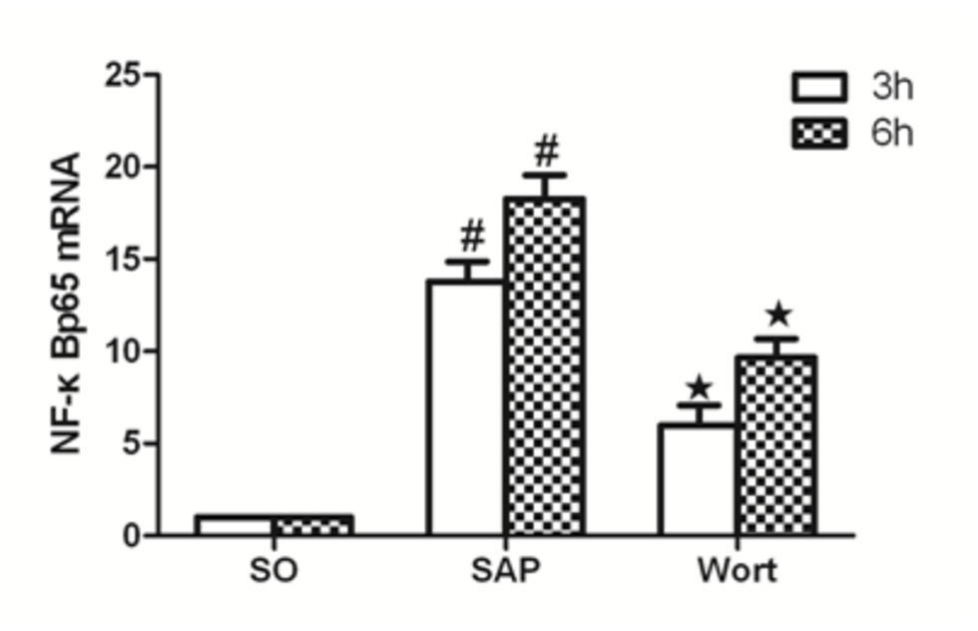
NF-κBp65 mRNA expression in each group at the 3 h and 6 h time point. ^*#*^ *P*<0.05 vs. SO group; ^★^ *P*<0.05 vs. SAP group.

**Figure 9:**
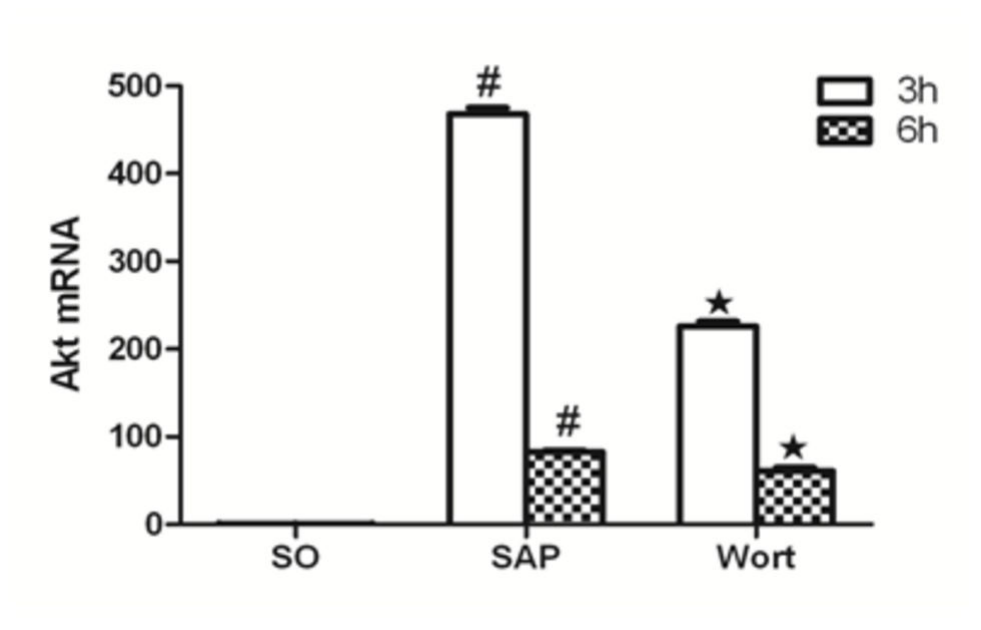
Akt mRNA expression in each group at the 3 h and 6 h time point. ^*#*^ *P*<0.05 vs. SO group; ^★^ *P*<0.05 vs. SAP group.

The level of Akt, p38MAPK, and NF-κBp65 mRNA expression from the control pancreas was assessed (Figure 7, Figure 8, and Figure 9). Compared with the SO group, Akt, p38MAPK, and NF-κBp65 mRNA markedly increased in pancreatic tissues of SAP rats at the 3 h and 6 h time points (P < 0.05). The wortmannin treatment decreased the expression of Akt, p38MAPK, and NF-κBp65 mRNA in the pancreas. At the 3 h time point for the W group, Akt, p38MAPK, and NF-κBp65 markedly decreased in the pancreatic tissues of rats. As NF-κBp65 and p38MAPK are downstream signaling molecules of inflammatory reaction associated with SAP, these results strongly suggest that wortmannin attenuated the sodium taurocholate-induced expression of Akt, p38MAPK, and NF-κBp65 mRNA in the pancreas. 

### p-Akt/Akt, p-p38MAPK/p38MAPK, and p-NF-κBp65/NF-κBp65 protein expression

**Figure 10:**
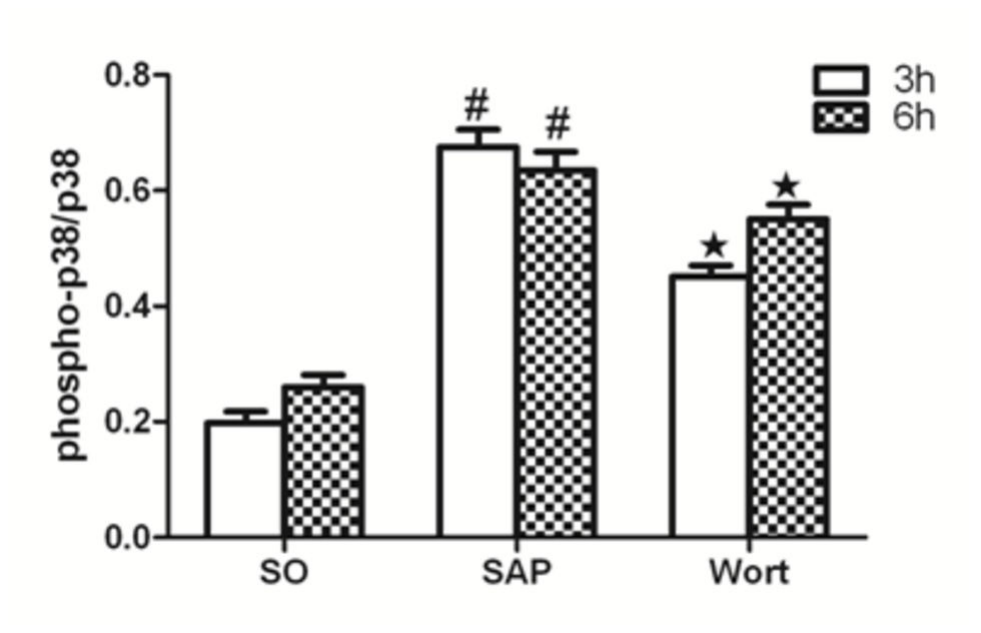
p-p38MAPK/p38MAPK protein expression in each group at the 3 h and 6 h time point. ^*#*^ *P*<0.05 vs. SO group; ^★^ *P*<0.05 vs. SAP group.

**Figure 11:**
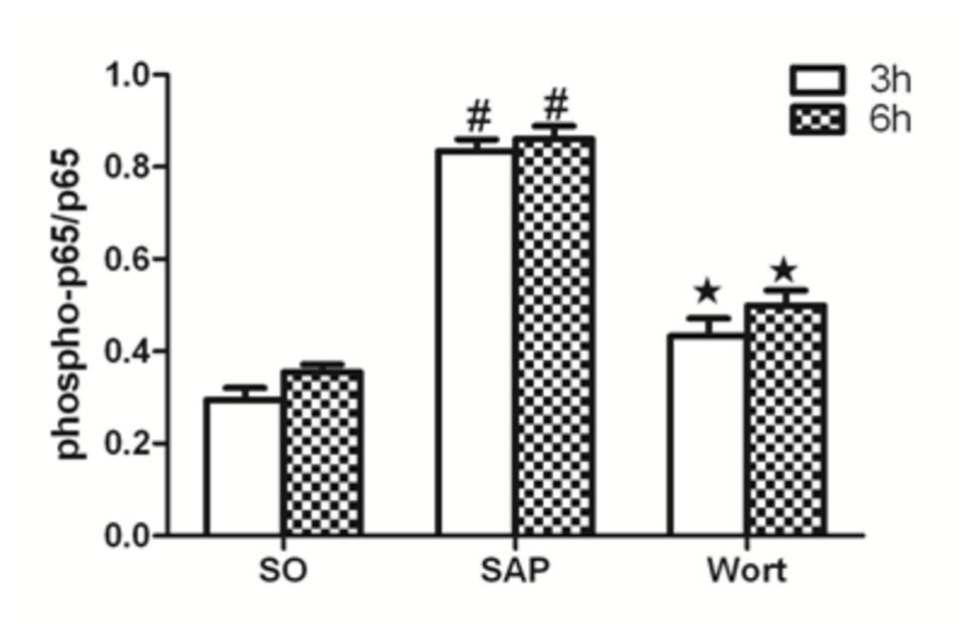
p-p65/p65 protein expression in each group at the 3 h and 6 h time point. ^*#*^ *P*<0.05 vs. SO group; ^★^ *P*<0.05 vs. SAP group.

**Figure 12:**
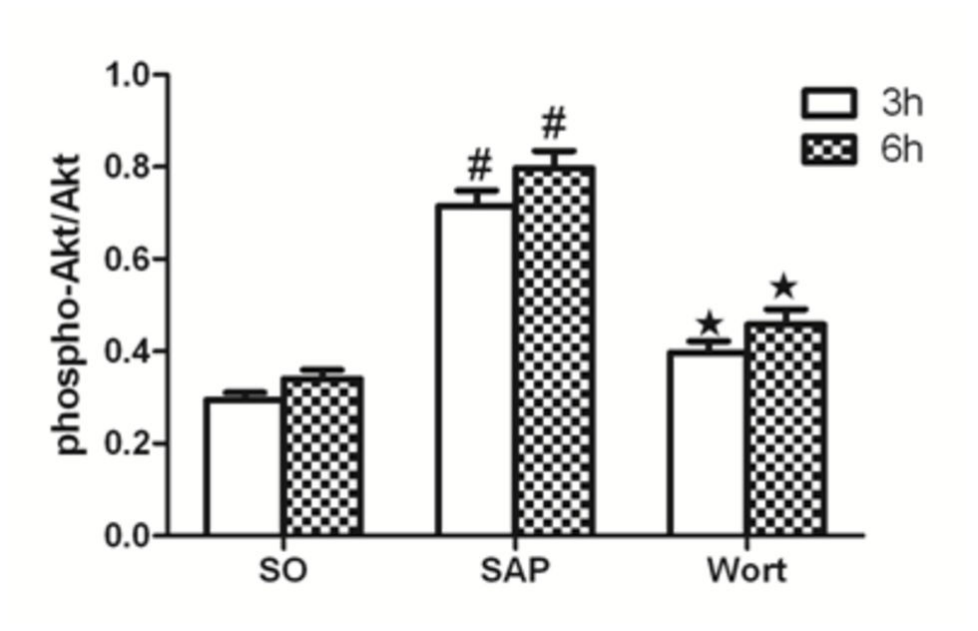
p-Akt/Akt protein expression in each group at the 3 h and 6 h time point. ^*#*^ *P*<0.05 vs. SO group; ^★^ *P*<0.05 vs. SAP group.

**Figure 13:**
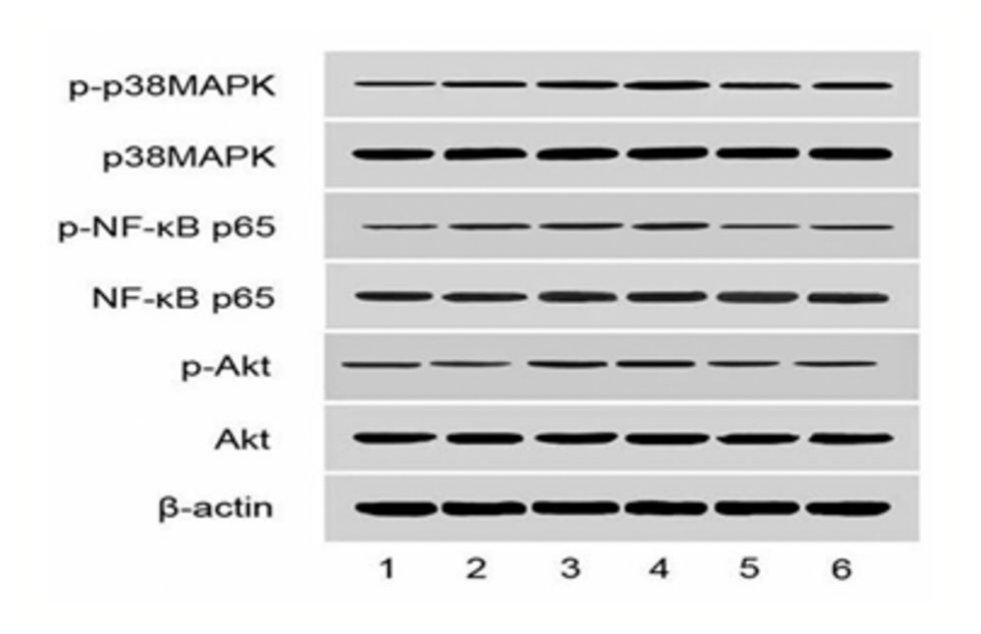
Western blot analysis of each group at the 3 h and 6 h time point. 1:SO group at 3h; 2: SO group at 6h; 3:SAP group at 3h; 4: SAP group at 6h; 5: W group at 3h; 6: W group at 6h.

Akt, NF-κBp65, and p38MAPK are downstream signaling molecules in inflammation and the activation of these molecules are implicated in pancreatic acinar cell injury and inﬂammatory reactions associated with SAP. The protein expression of p-Akt/Akt, p-p38MAPK/p38MAPK, and p-NF-κBp65/NF-κBp65 in SAP groups were markedly upregulated at the 3 h and 6 h time points compared to the SO group. In contrast, the wortmannin treatment resulted in the decreased protein expression of p-Akt/Akt, p-p38MAPK/p38MAPK, and p-NF-κBp65/NF-κBp65 in the pancreas. These results further suggested that wortmannin could attenuate the severity of SAP (Figure 10, Figure 11, Figure 12, and Figure 13).

## Discussion

Sodium taurocholate-induced pancreatitis in mice or rats is the most well-characterized model of pancreatitis. This model has been extensively employed for probing events that may be critical to the evolution of pancreatitis [11]. These pathological changes include the colocalization of lysosomal hydrolases with digestive enzyme zymogens in cytoplasmic vacuoles, the intra-acinar cell activation of trypsinogen, and the activation of NF-κB and p38MAPK [12],[13]. The mechanisms responsible for these events, as well as the relationship between each of these changes and the subsequent development of cell injury and pancreatitis, are incompletely understood.

Wortmannin, which is known to inhibit PI3K/Akt, markedly reduced the subcellular redistribution of cathepsin B in the AP rat model [14]. The previous paper also found that doses of wortmannin markedly reduced trypsinogen activation *in vitro* and *in vivo* during the early stages of secretagogue-induced pancreatitis [15]. Wortmannin administration in this study markedly reduced the severity of secretagogue-induced pancreatitis when evaluated at 30 min because it reduced the extent of pancreatic edema, neutrophil sequestration within the pancreas, extent of acinar cell necrosis, and magnitude of blood hyper-amylasemia and tissue myeloperoxidase [16]. These observations are compatible with the conclusion that PI3K/Akt inhibition protects against pancreatitis by preventing the intracellular colocalization of lysosomal hydrolases with digestive enzyme zymogens and the intracellular activation of trypsinogen [17]. Wortmannin is an effective treatment for inhibiting the intra-pancreatic activation of trypsinogen and regulating the severity of acute pancreatitis[18].

In this study, we investigated the impact of wortmannin on severe acute pancreatitis when applied to the SAP model of STC retrograde injection into the pancreatic duct. The results showed that wortmannin not only decreased the severity of pancreatic damage but also inhibited the release of the proinflammatory cytokines TNF-α, IL-1β, and IL-6 in sera, which coincided with histological changes in the pancreas. These findings suggest that wortmannin acts as a therapeutic drug in SAP rats. To further explore the role of PI3K/Akt in SAP, we used an *in vivo* study to classify the Akt expression. The reduced activation of Akt was found in pancreatic tissues of SAP rats at 3 h and 6 h after treatment with wortmannin. Since the low concentrations of wortmannin used in our studies were generally believed to specifically inhibit PI3K activity, these observations support our conclusion that wortmannin prevents inflammation activation *in vivo* by inhibiting the PI3K/Akt signaling pathway [19,20].

Our previous study confirmed that the activation of NF-κB and p38MAPK strongly responds to the drug pioglitazone during pancreatic injury and that activation of these molecules plays a key role not only in the release of inflammatory factors, but also in increasing neutrophil infiltration of the pancreas [7]. In this study, we analyzed the phosphorylation of Akt, NF-κB, and p38MAPK in SAP rats after intraperitoneally administering wortmannin. The results showed that the expression of p-Akt/Akt, p-p38MAPK/p38MAPK, and p-NF-κBp65/NF-κBp65 increased in SAP rats compared with the SO group at the 3 h and 6 h time points. Furthermore, after treatment with wortmannin in SAP rats, p-Akt/Akt, p-p38MAPK/p38MAPK, and p-NF-κBp65/NF-κBp65 constantly decreased and paralleled the level of pathological injury to the pancreas. Based on the above data, we found that inhibition of the PI3K/Akt pathway dramatically downregulated the intracellular signaling pathway of the transcription factor NF-κB, p38MAPK, and cytokines in SAP rats. Recent studies have shown that PI3K/Akt is an important mediator for regulating NF-κB-dependent gene expression in various cell types. It has also been well-established that inflammatory responses in acute pancreatitis are highly dependent on the activation of the transcription factor NF-κB, which plays an important role in the regulation of several genes [21]. A recent study also showed that the PI3K/Akt pathway could regulate NF-κB nuclear activity by affecting its interaction with the cAMP response element-binding protein and CREB-binding protein [22,23]. In addition, Akt reduction was also shown to inhibit MAP3Ks, ASK1, and Raf-1, molecules required for the activation of p38MAPK [24]. Other studies support our observation that the inhibition of PI3K/Akt signaling alleviates the secretion of inflammatory factors through downregulating p38MAPK [25].

A previous study reported that wortmannin can reduce trypsinogen activation that occurs at the early stages (within 30 min) in two different experimental models of rodent acute pancreatitis (caerulein-induced and taurocholate-induced pancreatitis) [16]. This study also suggested that the administration of wortmannin could not alter NF-κB activation within 30 min of caerulein-induced pancreatitis. Therefore, the paper concluded that a wortmannin alleviated the severity of acute pancreatitis via inhibiting trypsinogen activation at the early stages (30 min). However, the activation of NF-κB and p38MAPK activation with time was not investigated in taurocholate-induced pancreatitis. Interestingly, in our research, wortmannin could decrease NF-κB and p38MAPK expression at the later stages (3h, 6h) of taurocholate-induced pancreatitis. Therefore, our results differ from previous reports by demonstrating that wortmannin alleviates the severity of acute pancreatitis via inhibiting NF-κB and p38MAPK activation at the later stages of taurocholate-induced pancreatitis. The mechanisms induced by PI3K/Akt signaling pathway to regulate NF-κB and p38MAPK activity in SAP rats are currently under investigation and further in-depth studies are necessary to interpret its therapeutic effects.

## Conclusion

In summary, our findings show that the PI3K/Akt inhibitor wortmannin decreases inflammatory cytokines in SAP. Moreover, the mechanisms of PI3K/Akt inhibitor may be the regulatory role of PI3K/Akt signaling pathway mediated by a reduction in the activation of NF-κB and p38MAPK activity in SAP rats and down-regulation of transcription of NF- κB-dependent pro-inflammatory genes, including TNF-α, IL-1β and IL-6. Taken together, our results suggest that the pharmacological activation of the PI3K/Akt pathway may aid in the development of new therapeutics to limit inflammation in SAP diseases.
